# Supplementary material for: GCN5L1 modulates cross-talk between mitochondria and cell signaling to regulate FoxO1 stability and gluconeogenesis
Source: Nat Commun. 2017 Sep 12;8:523. doi: 10.1038/s41467-017-00521-8 (PMC5595826; doi:10.1038/s41467-017-00521-8)

File name: Supplementary Information
Description: Supplementary Figures

File name: Supplementary Data 1
Description: Phosphorylated peptides from WT and GCN5L1 LKO hepatocytes overexpressed Flag-FoxO1 (Related to Supplementary Figure 4a).

File name: Supplementary Data 2
Description: Representative spectra and peak lists of phosphorylated peptides from WT and GCN5L1 LKO hepatocytes overexpressed Flag-FoxO1

File Name: Peer Review File
Description:

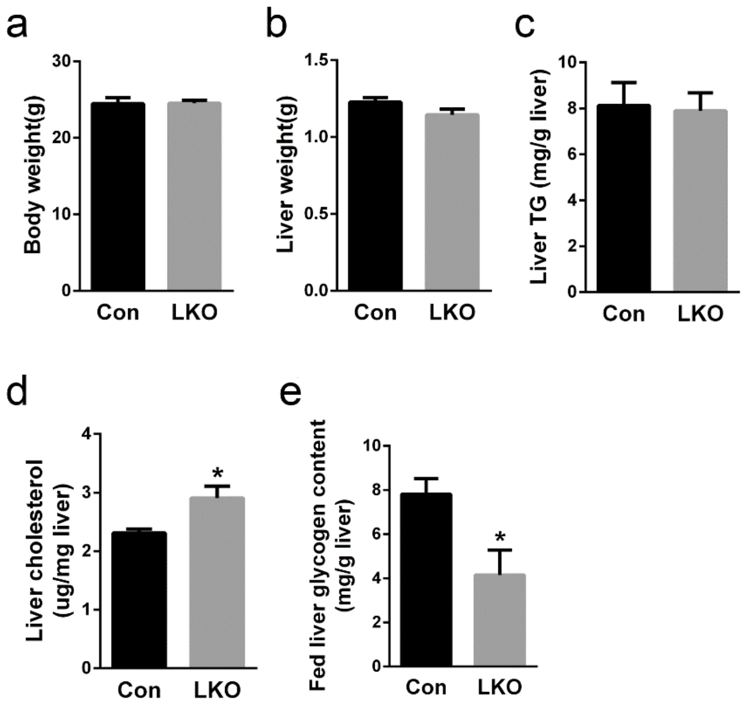

Supplementary Figure 1: Basal Phenotypes of GCN5L1 LKO Mice.
Body weight (a) and liver weight (b) of male mice at 8-10 weeks of age. (c) Liver triglyceride content. (d)Liver cholesterol content and (e) Liver glycogen content of these mice. n=6 for each group. Values are expressed as mean ± s.e.m. *P<0.05; **P<0.01 by Student’s *t*-test.

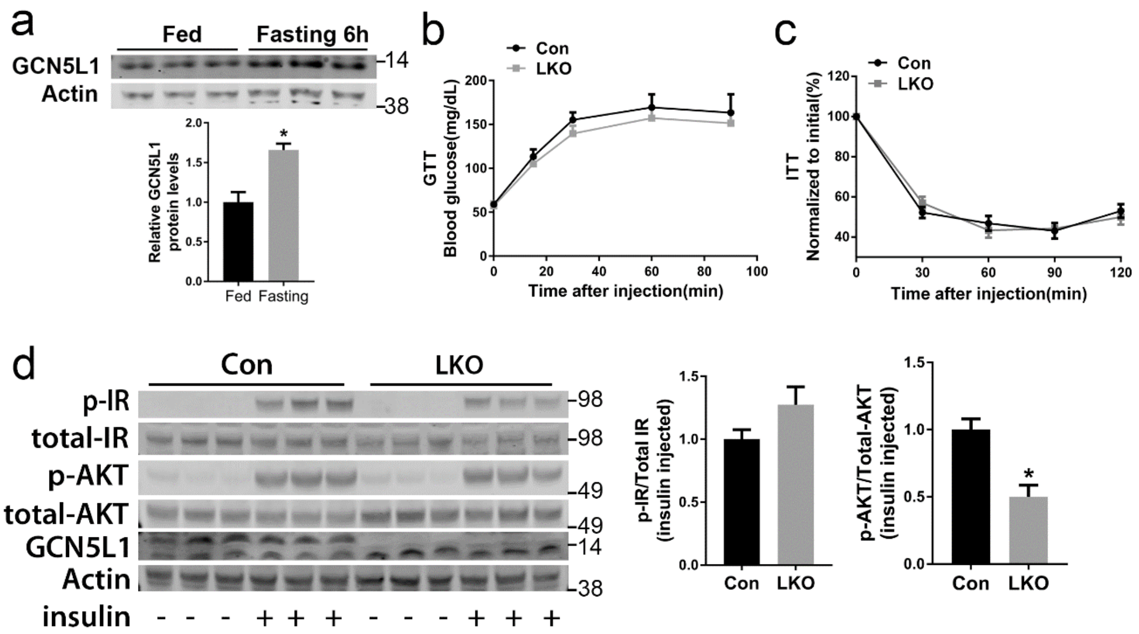

Supplementary Figure 2: Glucose and insulin tolerance phenotypes of GCN5L1 LKO mice. 9-10 weeks male mice fed with normal chow were studied. (a) GCN5L1 protein levels under fed and 6h fasting conditions. 3 representative mice were shown for immunoblotting in each condition. (n=5 for each condition. (b) Glucose tolerance test was performed on overnight fasted mice. (n=7 for each group). (c) Insulin tolerance test was performed 4 hour fasted mice (n=10 for Con, n=12 for LKO). (d) In vivo insulin signaling pathway was analyzed by immunoblotting phosphorylation of IR and AKT in liver tissues with/without insulin injection. Quantification of insulin injected p-IR or p-AKT normalized to total-IR or total-AKT, relative to insulin injected Con group (n=3 for each group). Values are expressed as mean ± s.e.m. *P<0.05 by Student's *t*-test.

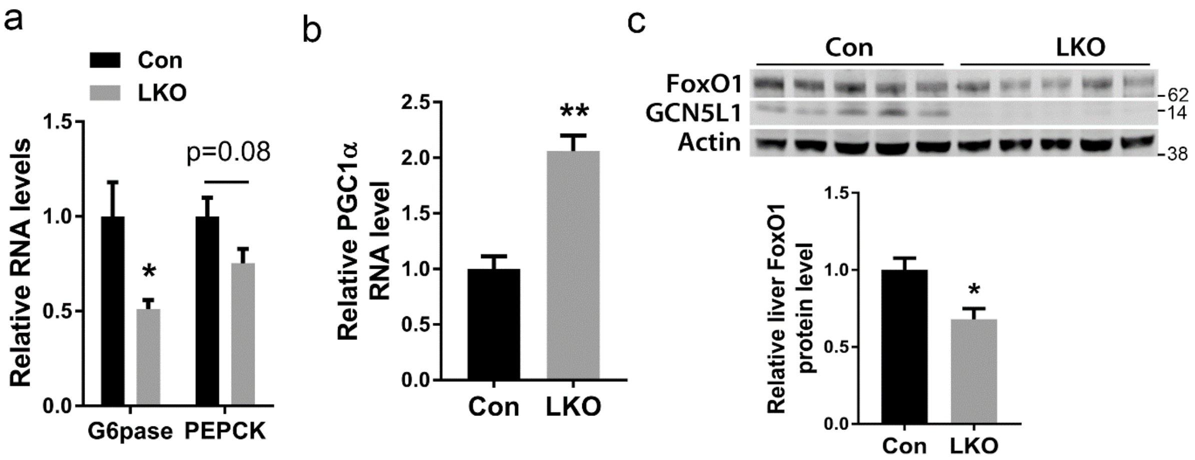

Supplementary Figurer 3: Gluconeogenic transcripts and FoxO1 protein levels are decreased in GCN5L1 LKO livers.

9-12 weeks male mice were fasted overnight and used for studies. (**a**) Liver RNA levels of G6Pase and PEPCK (n=6 for each group). (b) Liver RNA level of PGC1α (n=6 for each group). (c) Liver FoxO1 protein levels were assayed by immunoblotting. Quantification of FoxO1 normalized to β-Actin, relative to Con group (n=5 for each group). Values are expressed as mean ± s.e.m. *P<0.05; **P<0.01 versus Con groups, by Student's *t*-test.

a

Annotated Sequence	Phosphorylation Site	Rel. Occupancy
[R].TSSNASTISGRLSPIMTEQDDLGDGDVHSLVYPPSAAK.[M]	S322; S326	2.70E+08
[R].TSSNASTISGRLSPIMTEQDDLGDGDVHSLVYPPSAAK.[M]	S319/T320	2.80E+08
[R].TSSNASTISGRLSPIMTEQDDLGDGDVHSLVYPPSAAK.[M]	T320/S319	1.55E+09
[R].LSPIMTEQDDLGDGDVHSLVYPPSAAK.[M]	S326	2.50E+07
[K].ASLQSGQEGPGDSPGSQFSKWPASPGSHSNDDFDNWSTFRPR.[T]	S290; S295	2.20E+08
[K].ASLQSGQEGPGDSPGSQFSKWPASPGSHSNDDFDNWSTFRPR.[T]	S295	1.30E+08
[K].WPASPGSHSNDDFDNWSTFRPR.[T]	S295; S298	1.40E+08
[K].WPASPGSHSNDDFDNWSTFRPR.[T]	S295	3.45E+08
[K].ELLTSDSPPHNDIMSPVDPGVAQPNSR.[V]	S465; S467	3.80E+07
[K].ELLTSDSPPHNDIMSPVDPGVAQPNSR.[V]	S467; S475	1.50E+08
[K].ELLTSDSPPHNDIMSPVDPGVAQPNSR.[V]	S465	1.91E+08
[K].ELLTSDSPPHNDIMSPVDPGVAQPNSR.[V]	S467	7.74E+08
[K].ASLQSGQEGPGDSPGSQFSK.[W]	S284	2.10E+07

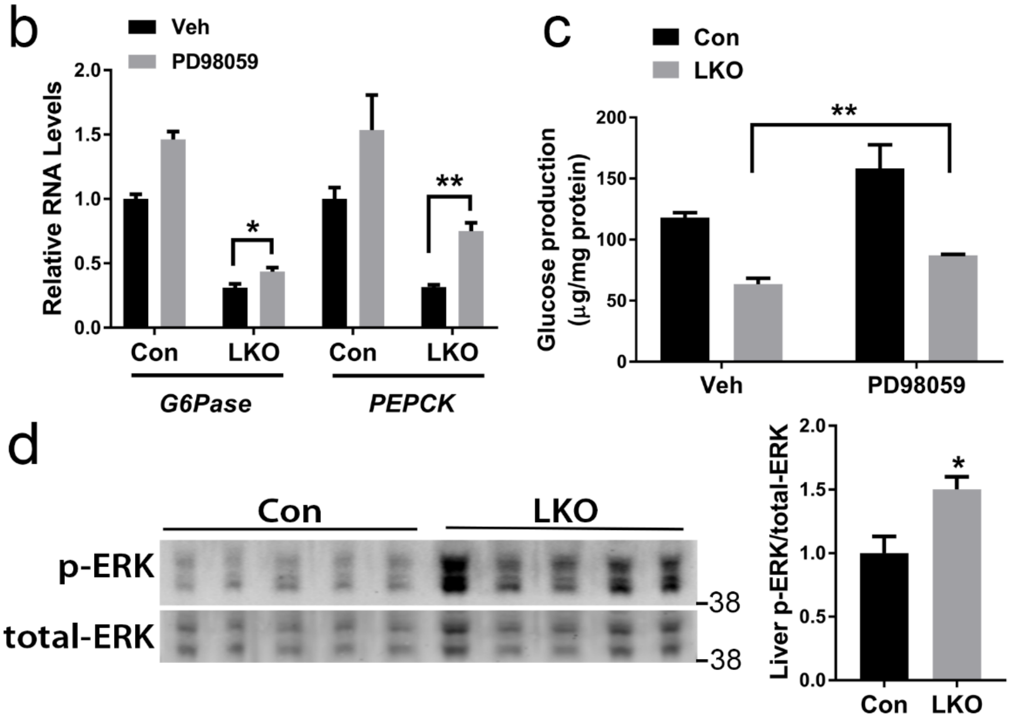

Supplementary Figure 4: GCN5L1 LKO controls FoxO1 phosphorylation and gluconeogenesis via activation of ERK.

(a) FoxO1 phosphorylation sites and fold change of area in control and GCN5L1 LKO primary hepatocytes. Primary hepatocytes were infected with adenoviral expression of Flag-FoxO1, phosphorylation sites of FoxO1 was analyzed by immunoprecipitation and mass spectrometry. Area ratio implicates the fold change of the AUC of individual sites in LKO divided by AUC in Con. The ratios were not calculated when the peptide phosphorylation residues were below the level of detection (n.d. – not detected). Known phosphorylated sites of FoxO1 by ERK/p38 were

marked in red. (b and c) Primary hepatocytes were isolated and incubated with PD98059 (PD) for 8 hours, RNA levels of G6Pase and PEPCK were analyzed by real-time PCR (b). (n=3 of independent experiments). Glucose production as assayed by secretion into the media from these cells(c). (n=3 of independent experiments). (d) Liver tissues from overnight fasting male mice were analyzed for phosphorylation of ERK. Quantification of p-ERK normalized to total-ERK, relative to Con group (n=5 for each group). Values are expressed as mean ± s.e.m. *$P<0.05$; **$P<0.01$ by Student's *t*-test.

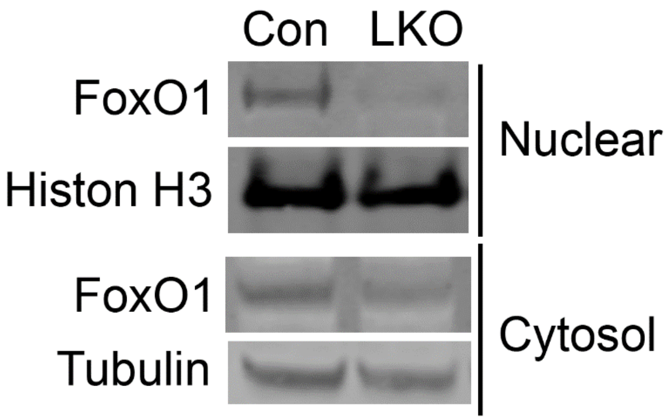

Supplementary Figure 5. The nuclear sequestration of FoxO1 is diminished in GCN5L1 LKO.

Nuclear and cytosolic fraction were extracted from primary hepatocytes, FoxO1 protein levels were analyzed by immunoblotting. Histon H3 serves as nuclear marker, and tubulin serves as cytosolic marker.

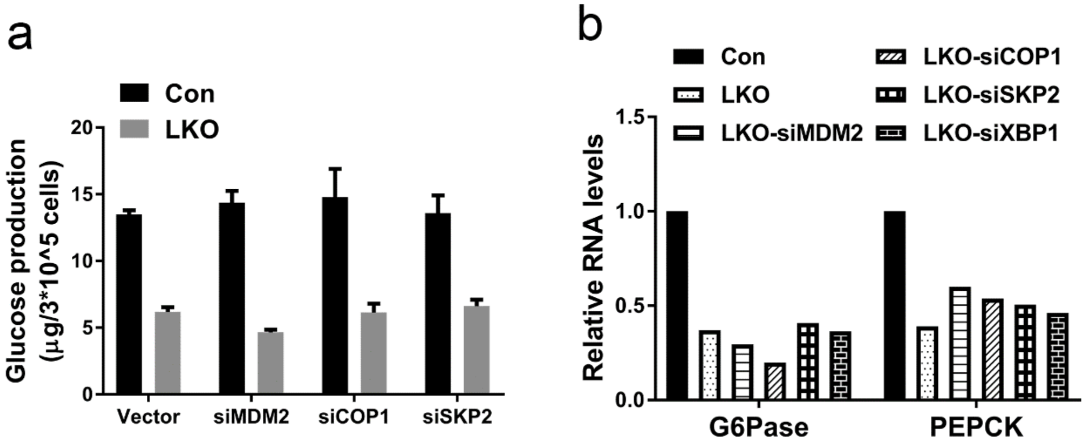

Supplementary Figure 6. Knockdown known FoxO1 degrading proteins did not restore gluconeogenesis in GCN5L1 LKO hepatocytes.

Primary hepatocytes were isolated from control and GCN5L1 LKO mice, knockdown of MDM2, COP1, SKP2 and XBP1 with siRNA transfection. Glucose production was measured in these cells(a). RNA levels of G6Pase and PEPCK were analyzed by real-time PCR(b).

Supplementary Figure 7. Blots correspond to the immunoblotting graphs shown in the main manuscript and supplementary.

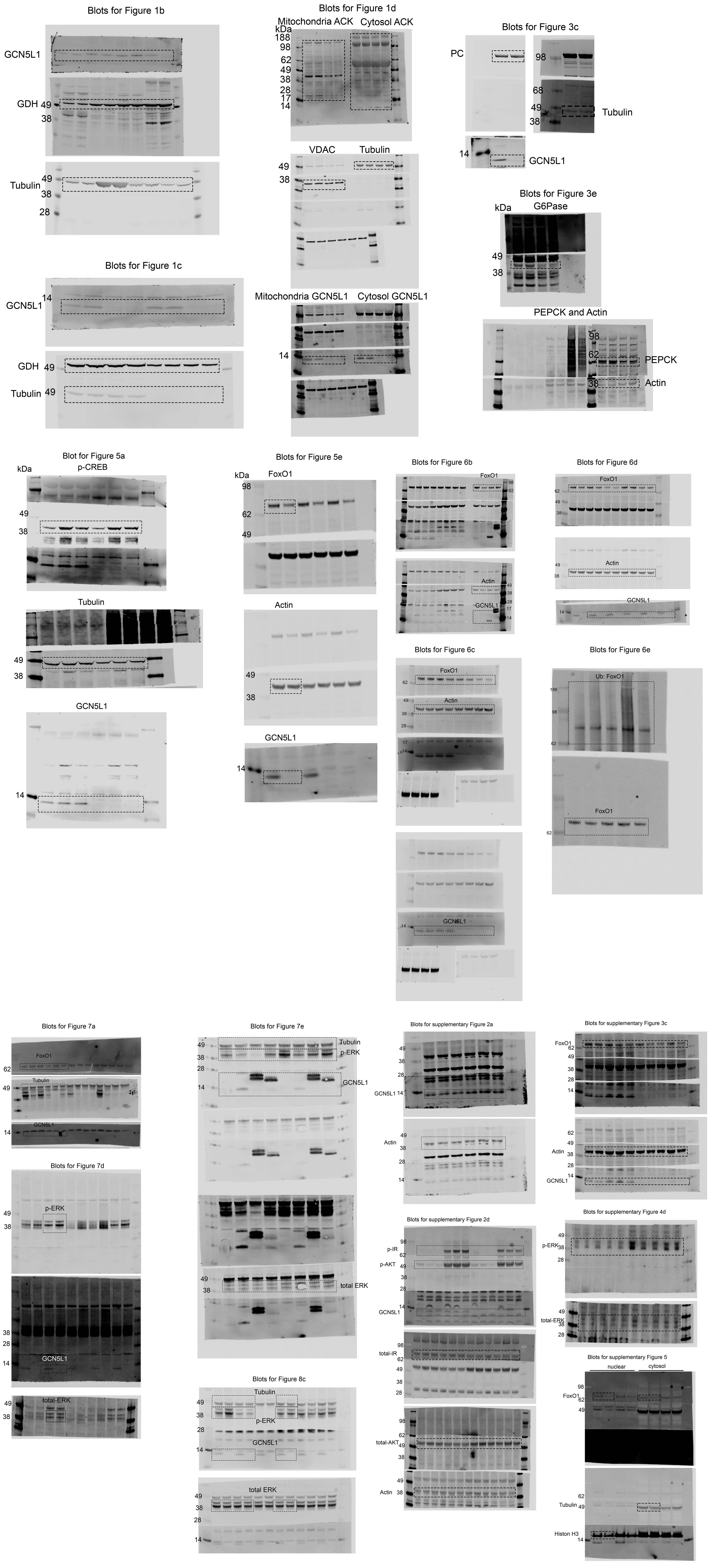

Supplement: Supplementary file 1 — Supplementary Information [file 41467_2017_521_MOESM1_ESM.pdf]
