# Supplementary material for: GCN5L1 modulates cross-talk between mitochondria and cell signaling to regulate FoxO1 stability and gluconeogenesis
Source: Nat Commun. 2017 Sep 12;8:523. doi: 10.1038/s41467-017-00521-8 (PMC5595826; doi:10.1038/s41467-017-00521-8)
Supplement: Supplementary file 3 — Supplementary Data 2 [file 41467_2017_521_MOESM3_ESM.docx]

Representative spectra and peak lists of phosphorylated peptides from WT and GCN5L1 LKO hepatocytes overexpressed Flag-FoxO1

Q9R1E0 [270-291] [K].KKASLQSGQEGPGDSPGSQFSK.[W] 1xPhospho [S]

Q9R1E0 [314-351] [R].TSSNASTISGRLSPIMTEQDDLGDGDVHSLVYPPSAAK.[M] 2xPhospho [T/Y/S]; 1xOxidation [M16]

Q9R1E0 [314-351] [R].TSSNASTISGRLSPIMTEQDDLGDGDVHSLVYPPSAAK.[M] 2xPhospho [S/T]

Q9R1E0 [314-351] [R].TSSNASTISGRLSPIMTEQDDLGDGDVHSLVYPPSAAK.[M] 1xPhospho [T/S]; 1xOxidation [M16]

Q9R1E0 [314-351] [R].TSSNASTISGRLSPIMTEQDDLGDGDVHSLVYPPSAAK.[M] 1xPhospho [S/T/Y]

Q9R1E0 [178-189] [R].LTLSQIYEWMVK.[S] 1xPhospho [S4]

Q9R1E0 [325-351] [R].LSPIMTEQDDLGDGDVHSLVYPPSAAK.[M] 1xPhospho [S2]

Q9R1E0 [272-313] [K].ASLQSGQEGPGDSPGSQFSKWPASPGSHSNDDFDNWSTFRPR.[T] 2xPhospho [S19; S24]

Q9R1E0 [272-313] [K].ASLQSGQEGPGDSPGSQFSKWPASPGSHSNDDFDNWSTFRPR.[T] 1xPhospho [S24]

Q9R1E0 [177-189] [K].RLTLSQIYEWMVK.[S] 1xPhospho [Y/S/T]

Q9R1E0 [292-313] [K].WPASPGSHSNDDFDNWSTFRPR.[T] 2xPhospho [S4; S7]

Q9R1E0 [292-313] [K].WPASPGSHSNDDFDNWSTFRPR.[T] 1xPhospho [S4]


Q9R1E0 [461-487] [K].ELLTSDSPPHNDIMSPVDPGVAQPNSR.[V] 2xPhospho [S7; S]; 1xOxidation [M14]

Q9R1E0 [461-487] [K].ELLTSDSPPHNDIMSPVDPGVAQPNSR.[V] 2xPhospho [S7; S]

Q9R1E0 [461-487] [K].ELLTSDSPPHNDIMSPVDPGVAQPNSR.[V] 1xPhospho [T/S]; 1xOxidation [M14]

Q9R1E0 [461-487] [K].ELLTSDSPPHNDIMSPVDPGVAQPNSR.[V] 1xPhospho [S/T]


Q9R1E0 [272-291] [K].ASLQSGQEGPGDSPGSQFSK.[W] 1xPhospho [S13]


Q9R1E0 [271-291] [K].KASLQSGQEGPGDSPGSQFSK.[W] 1xPhospho [S14]

Q9R1E0 [314-351] [R].TSSNASTISGRLSPIMTEQDDLGDGDVHSLVYPPSAAK.[M] 3xPhospho [S3; S6; S9]

Q9R1E0 [444-487] [K].SSYGGLNQYNCAPGLLKELLTSDSPPHNDIMSPVDPGVAQPNSR.[V] 1xCarbamidomethyl [C11]; 1xPhospho [S/T/Y]
